# Cynaroside ameliorates TNBS-induced colitis by inhibiting intestinal epithelial cell apoptosis via the PI3K/AKT signalling pathway

**DOI:** 10.3389/fphar.2024.1496068

**Published:** 2025-01-20

**Authors:** Ju Huang, Jing Li, Zhijun Geng, Lixia Yin, Minzhu Niu, Qingqing Li, Xinyue Liu, Xinke Cheng, Xiaofeng Zhang, Xue Song, Yueyue Wang, Lian Wang, Lugen Zuo, Jianguo Hu

**Affiliations:** ^1^ Department of Clinical Laboratory, First Affiliated Hospital of Bengbu Medical University, Bengbu, Anhui, China; ^2^ Anhui Province Key Laboratory of Basic and Translational Research of Inflammation-Related Diseases, First Affiliated Hospital of Bengbu Medical University, Bengbu, Anhui, China; ^3^ Department of Central Laboratory, First Affiliated Hospital of Bengbu Medical University, Bengbu, Anhui, China; ^4^ Department of Clinical Laboratory, The Third the People’s Hospital of Bengbu, Bengbu, Anhui, China

**Keywords:** Crohn’s disease, cynaroside, IECs apoptosis, colonic organoids, PI3K/AKT

## Abstract

**Background and aims:**

Patients with Crohn’s disease (CD) exhibit excessive apoptosis of intestinal epithelial cells (IECs), which contributes to damage to the intestinal barrier structure and function, thereby playing a role in the progression of colitis. Preventing IEC apoptosis and protecting the intestinal barrier are critical to alleviating colitis. Natural plant monomers have been reported to possess multiple pharmacological properties, particularly with the potential to treat CD. This study focuses on Cynaroside (Cyn) to explore its effect on IEC apoptosis and evaluate its pharmacological impact on the intestinal barrier and colitis.

**Methods:**

The 2,4,6-trinitrobenzenesulfonic acid (TNBS)-induced CD-like colitis mice model was employed in this study. We assessed the therapeutic effect of Cyn on CD-like colitis by evaluating the disease activity index (DAI), body weight changes, intestinal tissue pathological damage, and inflammatory factor levels. Immunofluorescence and Western blotting were used to detect the expression and localization of tight junction (TJ) proteins, allowing us to analyze the intestinal barrier structure. The function of the intestinal barrier was examined using FITC-dextran (FD4), TEER values, and bacterial translocation. Network pharmacology enrichment analysis revealed that Cyn could inhibit cell apoptosis. We also explored the effect and underlying mechanism of Cyn in inhibiting IEC apoptosis on intestinal barrier function and colitis using both the TNF-α-induced colonic organoid model and the TNBS-induced mouse model.

**Results:**

Our findings show that Cyn significantly alleviates TNBS-induced colitis symptoms in mice, as evidenced by reduced body weight loss, colon shortening, DAI score, colon histopathology score, and lower levels of inflammatory factors (IL-1β, TNF-α, and IL-6) compared to the model group. Additionally, the Cyn intervention group showed significant improvements in both the intestinal barrier structure (elevated tight junction protein levels and proper localization) and function (reduced serum FD4 levels, increased intestinal TEER, and decreased bacterial translocation rates in mesenteric lymph nodes [MLNs] and livers). Combining network pharmacology prediction analysis with our validation data from animal models and colonic organoids, we demonstrated that Cyn significantly inhibits IEC apoptosis, as indicated by a decrease in the proportion of TUNEL-positive cells and changes in apoptosis-related protein levels. KEGG enrichment analysis and signaling pathway intervention experiments confirmed that Cyn inhibits the activation of PI3K/AKT signaling.

**Conclusion:**

Cyn inhibits IEC apoptosis by blocking the PI3K/AKT signaling pathway, which is the primary mechanism underlying its protective effects on the intestinal barrier and its ability to improve CD-like colitis. This study also supports the potential of the Chinese medicine monomer Cyn as a promising therapeutic agent for the treatment of CD.

## 1 Introduction

Crohn’s disease (CD) is a chronic inflammatory bowel disease of unknown etiology, characterized by recurrent episodes that damage the intestinal tract and significantly reduce the patient’s quality of life (Torres et al., 2017; Roda et al., 2020). Currently, clinical management of CD relies heavily on pharmacological treatments, yet long-term disease remission remains challenging. Moreover, patients frequently experience toxic side effects and drug tolerance, highlighting the urgent need for safe and effective therapeutic options (Cushing and Higgins, 2021; Parigi et al., 2023). Impaired intestinal barrier function is not only a hallmark of CD but also a contributing factor to its progression. Therefore, targeting intestinal barrier permeability represents a promising therapeutic strategy for managing the disease (Chelakkot et al., 2018; Turpin et al., 2020). Increased intestinal permeability, accompanied by elevated intestinal inflammation, has been observed in CD. Studies indicate that specifically protecting the intestinal barrier function can lead to significant improvements in colitis (Zhang HX. et al., 2022; Zhuang et al., 2022; Zhao et al., 2023; Kaminsky et al., 2021). A single layer of columnar intestinal epithelial cells (IECs) plays a crucial role in maintaining the integrity of the epithelial barrier and serves as the structural foundation for intestinal barrier function (Chelakkot et al., 2018; Ngo et al., 2022; Untersmayr et al., 2022). In both CD patients and CD-like colitis mouse models, excessive IEC apoptosis and increased intestinal permeability have been observed (Tao et al., 2021; Wen et al., 2022; Zhang M. et al., 2024). Therefore, therapeutic strategies aimed at preventing IEC apoptosis may improve intestinal barrier function and reduce symptoms associated with intestinal inflammation.

Cynaroside (Cyn), also known as luteolin-7-O-glucoside (LUT-7G), is a flavonoid compound found in plants such as snow chrysanthemum and honeysuckle. Previous studies have confirmed that Cyn exhibits various pharmacological activities, including anti-apoptotic, antioxidant, anti-tumor, and anti-inflammatory effects (De Stefano et al., 2021; Bouyahya et al., 2023). It has been reported that Cyn inhibits *Staphylococcus* aureus-induced apoptosis in endometrial epithelial cells (Wang et al., 2018), prevents H2O2-induced apoptosis in H9c2 cells (Sun et al., 2011), and inhibits caspase-3 activation, mitochondrial dysfunction, and apoptosis triggered by cisplatin in HK-2 cells (Nho et al., 2018). These findings suggest that Cyn has significant anti-apoptotic properties. Furthermore, studies indicate that Cyn can be absorbed through the gastrointestinal tract (Zhang J. et al., 2022). Recently, Cyn has also been shown to alleviate methotrexate (MTX)-induced intestinal inflammation in rats and increase the number of intestinal goblet cells (Lang et al., 2024). However, the effects of Cyn on IEC apoptosis *in vivo* models of CD and its potential for treating CD-like colitis remain unclear.

The phosphatidylinositol-3-kinase/phosphatidylserine-threonine kinase serine-threonine kinase (PI3K/AKT) signaling pathway transduces signals from various receptors and regulates biological homeostasis by modulating cell survival, growth, proliferation, and apoptosis (Cantley, 2002). Liu et al. (Liu et al., 2020) reported that inhibiting the PI3K/AKT pathway improved the intestinal barrier and anti-inflammatory properties in colitis. Furthermore, the PI3K/AKT pathway is known to play a crucial role in regulating IEC apoptosis in colitis, and targeted inhibition of this pathway can prevent apoptosis induced by TNBS or inflammatory factors (Li et al., 2024; Zhang Z. et al., 2024).

In this study, we found that Cyn improves TNBS-induced CD-like colitis in mice and helps maintain both the structure and function of the intestinal barrier. More importantly, our results confirm that Cyn inhibits IEC apoptosis in both *in vivo* models and the TNF-α-induced colonic organoid apoptosis model, which is linked to the inhibition of PI3K/AKT signaling activation by Cyn. In summary, our study suggests that Cyn can improve TNBS-induced colitis by inhibiting IEC apoptosis and preserving the integrity of the intestinal barrier, offering a potential new approach for developing CD therapeutics and further expanding the pharmacological effects of Cyn.

## 2 Materials and methods

### 2.1 Animals and *in vivo* studies

A specific-pathogen-free (SPF) environment was maintained for male C57BL/6J mice, aged 6–8 weeks and weighing 20 ± 2 g. All animal experimental procedures were approved by the Bengbu Medical University Animal Ethics Committee ([2023] No. 572).

Five groups of C57BL/6J mice were randomly assigned: WT, TNBS, Cyn (5 mg/kg/d), Cyn (10 mg/kg/d), and Cyn (20 mg/kg/d) (Nho et al., 2018), with six mice in each group. The TNBS-induced colitis model was applied to the TNBS and Cyn groups, as follows: Mice were anesthetized via intraperitoneal injection of 1% sodium pentobarbital, placed in a head-down position, and treated with 100 μL of 2.5% TNBS solution (Sigma-Aldrich, St. Louis, Missouri, United States; 5% TNBS mixed with anhydrous ethanol 1:1) administered rectally via a thin polyethylene catheter. Following the model induction, mice in the Cyn groups were gavaged with 200 μL of Cyn (5, 10, or 20 mg/kg; Solarbio, Beijing, China; n = 6) daily for 6 days, while mice in the other groups were gavage with an equivalent volume of saline.

To investigate the role of the PI3K/AKT signaling pathway, C57BL/6J mice were randomly assigned to four groups: WT, TNBS, Cyn (10 mg/kg), and 740Y-P (a PI3K/AKT pathway agonist, MCE, NJ, United States). The first three groups were treated as described above, while mice in the 740Y-P group received TNBS modeling followed by continuous intraperitoneal injections of 740Y-P (10 mg/kg) (Zhang M. et al., 2024) and gavage of Cyn (10 mg/kg) for 6 days. Mice in each group consisted of six animals. Additionally, C57BL/6J mice were randomly divided into five groups: WT, TNBS, Cyn (10 mg/kg), Wor (Wortmannin, a PI3K/AKT pathway inhibitor, MCE), and Cyn + Wor. The first three groups were treated as described above, while mice in the Wor group were subjected to TNBS modeling and gavaged with Wortmannin (2 mg/kg) (Ihara et al., 2020) for 6 days. Mice in the Cyn + Wor group received TNBS modeling followed by continuous gavage of Wortmannin (2 mg/kg) and Cyn (10 mg/kg) for 6 days, with six mice per group.

The mice were euthanized on the sixth day following model induction. Blood, colon, liver, and mesenteric lymph node (MLN) samples were collected and preserved for further analysis.

### 2.2 Mouse colonic organoid culture and intervention

Following the methodology described by Kim et al. (2018) the colons of C57BL/6J mice were carefully excised using sterilized surgical scissors. The mesentery was removed and rinsed with cold D-PBS, after which the colon was cut into pieces. These pieces were washed with pre-cooled D-PBS at least 15 times to ensure tissue sterility. After cleaning, a dissociation reagent (GCDR, STEMCELL Technologies, Vancouver, CA) was added to isolate the crypts, which were then cultured in IntestiCult™ organoid growth media (STEMCELL Technologies).

To analyze the role of Cyn in apoptosis and barrier dysfunction in colonic organoids, we utilized a TNF-α-mediated IEC apoptosis model. After 10 days of cultivation, the organoids were randomly assigned to five groups: control, TNF-α, Cyn (25 μg/mL), Cyn (50 μg/mL), and Cyn (100 μg/mL). The three experimental groups were pretreated with Cyn (25, 50, or 100 μg/mL) for 4 hours (Sun et al., 2011), followed by a 24-h stimulation with 100 ng/mL of mouse recombinant TNF-α (Sigma-Aldrich) (Zhang X. et al., 2022). The method for MTT assessment of colonic organoids is provided in Supplementary Material S1.

To further investigate the regulatory mechanism of Cyn, we randomly assigned the organoids to four groups: control, TNF-α, Cyn, and 740Y-P. The first three groups were treated as described above, while the 740Y-P group was pretreated with Cyn (50 μg/mL) and 740Y-P (20 μM) for 4 hours, followed by a 24-h stimulation with 100 ng/mL of TNF-α. Additionally, we randomly divided the organoids into five groups: control, TNF-α, Cyn, Wor, and Cyn + Wor. The first three groups followed the same treatment protocol as the previous experiments; the Wor group was pretreated with Wortmannin (2 μM) (Wang et al., 2017) for 4 hours, followed by 24-h stimulation with 100 ng/mL of TNF-α. The Cyn + Wor group was pretreated with both Wortmannin (2 μM) and Cyn (50 μg/mL) for 4 hours, followed by stimulation with 100 ng/mL of TNF-α for 24 h.

### 2.3 Weight change

Body weights of the mice were measured and recorded daily.

### 2.4 Colon length

At the time of sample collection, the length of the mouse colon was measured and recorded.

### 2.5 Colitis symptom assessment

The Disease Activity Index (DAI) scoring method was used to assess the colitis symptoms, following the protocol outlined by Spencer et al. (Zuo et al., 2019). In brief, the severity of colitis was evaluated on a specified date by monitoring changes in body weight, the presence of occult or macroscopic rectal bleeding, and stool consistency, with scores ranging from 0 to 4. The DAI was calculated as the mean value of the various symptoms, which represents the level of colon inflammation. Occult blood was detected in the stool samples using a Hemoccult assay kit (Beckman Coulter, Inc., Fullerton, CA) (Zhao et al., 2007).

### 2.6 H&E and histological score

To assess morphological changes, mouse colon tissues were dehydrated, paraffin-embedded, sectioned, and stained with haematoxylin and eosin (H&E) (Appleyard and Wallace, 1995). Based on the H&E staining, the histological inflammation score of the colon tissue was evaluated on a five-point scale (0–4). The scoring criteria were as follows: 0 points for no inflammation and normal morphology; 1 point for mild inflammatory cell infiltration in the lamina propria; 2 points for mononuclear cell infiltration leading to crypt separation and mild mucosal hyperplasia; 3 points for extensive inflammatory cell infiltration causing disruption of the mucosal structure, loss of goblet cells, and marked mucosal hyperplasia; and 4 points for all the previous signs, along with crypt swelling and ulceration.

### 2.7 ELISA

Frozen colon tissues were ground and homogenized with 2 mL of saline containing protease inhibitors. The homogenate was centrifuged for 30 min to extract the supernatant. The protein levels of IL-1β, TNF-α, and IL-6 were then quantified using ELISA kits (BOSTER, Wuhan, China), following the manufacturer’s instructions.

### 2.8 qRT-PCR

The mRNA expression levels of proinflammatory mediators were measured by qRT-PCR, as previously described. Total RNA was extracted from the mouse colon mucosa tissue using TRIzol reagent. cDNA synthesis was performed using a cDNA synthesis kit (TaKaRa, Kusatsu, Japan), followed by reverse transcription. The mRNA was detected with the TB Green^®^ Premix Ex Taq™ II Detection System (TaKaRa), using primers specific to the target genes. The mouse-specific primer sequences used are listed in Supplementary Table S1.

### 2.9 *In vivo* FITC-dextran permeability assay

Mice were gavage with fluorescein isothiocyanate-labelled dextran (FITC-dextran 4, FD4, 4 kDa; Sigma-Aldrich) after a 4-h fasting period. Four hours later, the mice were euthanized, and 1 mL of blood was collected via heart puncture. The serum FITC-dextran fluorescence intensity was then measured.

### 2.10 Transepithelial electric resistance (TEER)

Fresh mouse colon tissues were thoroughly cleaned in phosphate-buffered saline (PBS) and placed in pre-warmed Krebs buffer. The tissues were cut into rectangles (2.8 mm by 11 mm) along the mesenteric axis. The samples were then inserted into the luminal system, and interepithelial resistance was measured according to the manufacturer’s guidelines.

### 2.11 Bacterial translocation

Liver and MLN samples from mice were aseptically collected for bacterial culture using previously described methods (Zuo et al., 2014). Briefly, two specimens of 0.1 g each were obtained from each tissue. Each specimen was homogenized in 0.9 mL of sterile saline. A 100 μL aliquot of the tissue homogenate was plated on MacConkey agar and incubated for 24 h. After incubation, the number of bacterial colony-forming units (CFUs) per Gram of tissue (Gram-negative bacteria, mainly Enterobacteriaceae) was counted. If 10^2^ CFUs or more were found per Gram, the culture result was considered positive.

### 2.12 Immunofluorescence

Mouse colon tissues and organoids were dehydrated, paraffin-embedded, and sectioned at 4 μm thickness. The sections were dewaxed in water and submerged in 0.01 M sodium citrate buffer, followed by heating for 60 min. After cooling, the sections were washed and blocked for 30 min with 5% BSA, Sigma-Aldrich. The sections were incubated overnight with primary antibodies against Claudin-1 (Abcam, Cambridge, UK, Cat #: ab15098) and ZO-1 (Thermo Fisher Scientific, MA, US, Cat #: 33–9100). The next day, the sections were treated with the appropriate fluorescent secondary antibodies for 2 h before nuclear staining.

### 2.13 Organoid permeability to FITC-dextran

The permeability of mouse colonic organoids to FITC-dextran was assessed as follows: 500 μL of Cultrex Organoid Harvesting Solution was used to dissolve the organoid matrix gel. After shaking for 20 min, the organoid solution was centrifuged at a low temperature for 5 min, and the supernatant was discarded. The remaining organoids were resuspended in FITC-dextran. Permeability was examined under a fluorescence microscope, and imaging results were obtained.

### 2.14 Network pharmacology analysis

The SMILES molecular formula and 2D structure diagram of Cynaroside were obtained from the PubChem database. Four additional databases were used to retrieve protein targets related to the drug: the PharmMapper database, Swiss Target Prediction database, SEA database, and Super-PRED database.

For disease gene targets, “Crohn’s disease” was used as a keyword to query five databases: the GeneCards database, PharmGKB database, Therapeutic Target Database (TTD), DrugBank database, and Online Mendelian Inheritance in Man (OMIM) database.

The drug and disease gene targets were imported into the Weishengxin website to generate Venn diagrams for both the drug and disease targets. Redundant targets were then removed from both datasets. The common gene targets between disease-related genes and drug prediction targets were identified using the VENNY website. These common target genes were further analyzed in the DAVID database for Gene Ontology (GO) functional annotation and Kyoto Encyclopedia of Genes and Genomes (KEGG) enrichment analysis. The results were visualized using the Weishengxin website.

### 2.15 TUNEL staining

After deparaffinization and hydration, paraffin sections of mouse colon tissues and organoids were incubated in labelling buffer at 37°C according to the instructions of the TMR (Red) TUNEL Apoptosis Detection Kit (Servicebio, Wuhan, China). DAPI staining was subsequently performed.

### 2.16 Western blotting

Frozen mouse colon mucosa tissues and mouse colonic organoids were harvested and thoroughly ground using a grinder. Proteins were extracted by adding lysate, and the supernatant was collected via centrifugation. Protein concentrations were determined using the BCA protein assay. Proteins were then denatured, electrophoresed, and transferred to a PVDF membrane. Primary antibodies targeting Bcl-2 (Cat #: ab182858), Bax (Cat #: ab32503), Cleaved-Caspase-3 (C-Cas3, Cat #: ab32042), Caspase-3 (Cas3, Cat #: ab32351), PI3K (Cat #: ab302958), p-PI3K (Cat #: ab278545), AKT (Cat #: ab8805), p-AKT (Cat #: ab38449), CREB (Cat #: ab32515), p-CREB (Cat #: ab32096), RXRA (Cat #: ab125001), and GAPDH (Cat #: ab8245) (Abcam) were incubated overnight. The secondary antibody was incubated for 2 hours the following day, and images were captured using an exposure apparatus. Relative protein amounts were then analyzed using ImageJ software.

### 2.17 Statistical analysis

Data were analyzed using SPSS 26.0 software. Group comparisons were performed using the unpaired *t*-test, and the results are expressed as the mean ± standard deviation (SD). Chi-square tests were used to analyze binary and categorical data, and P values less than 0.05 were considered statistically significant.

## 3 Results

### 3.1 Cyn relieves colitis in a mouse model of TNBS

**FIGURE 1:**
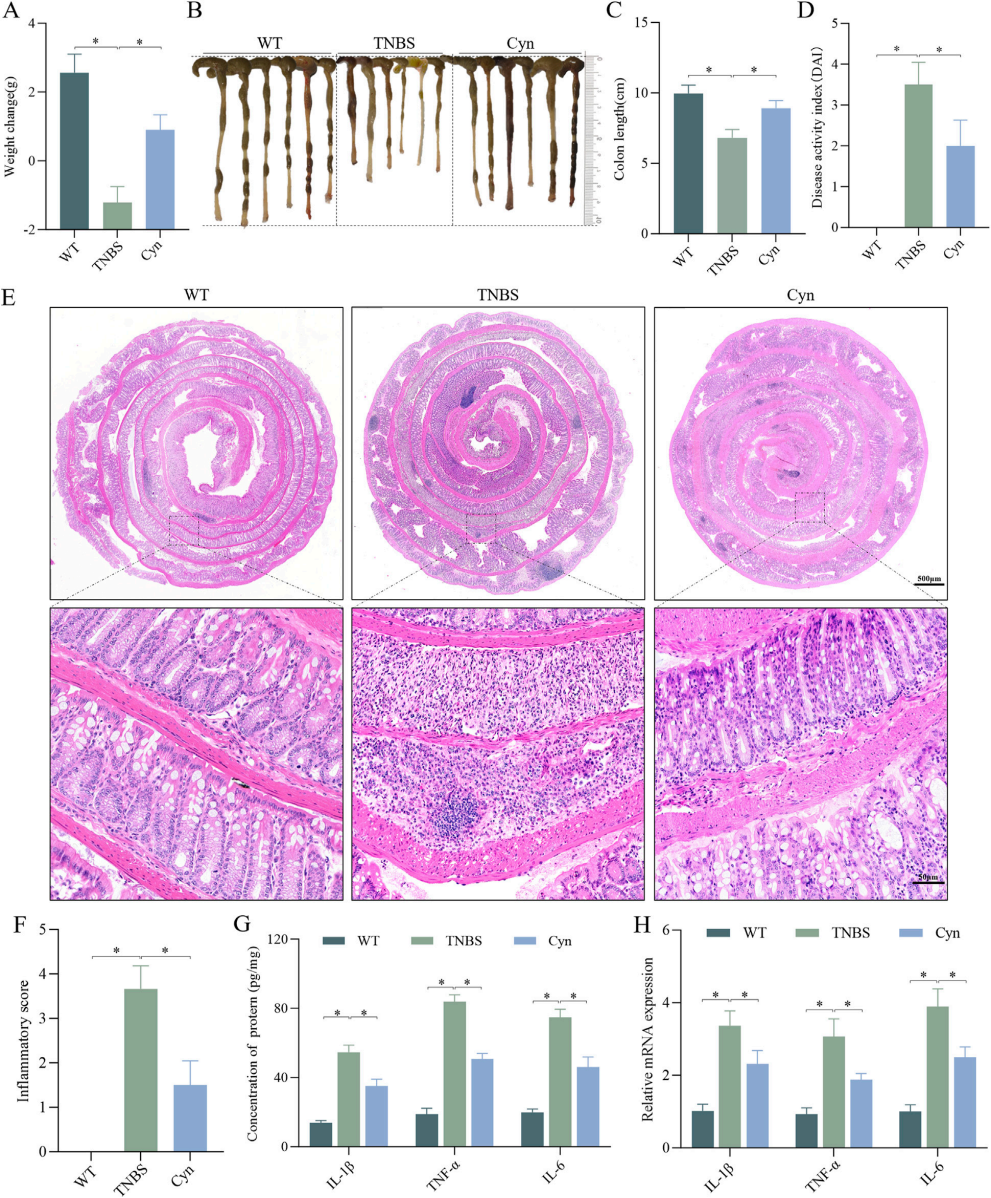
Cyn alleviates CD-like colitis in a TNBS-induced mouse model. **(A)** Changes in mouse body weight. **(B, C)** Mouse colon appearance and colon length. **(D)** DAI scores of the mice. **(E, F)** Colon inflammation scores and H&E staining for each mouse group. ELISA **(G)** and qRT‒PCR **(H)** analysis of proinflammatory cytokine expression in colon mucosa tissues. Data are presented as means ± standard deviations, n = 6, *p < 0.05.

To assess whether Cyn offers protective effects against colitis, we utilized a TNBS-induced CD-like colitis mouse model. A dose of 10 mg/kg of Cyn was selected based on the DAI score (Supplementary Figure S1). Compared to the TNBS-treated mice, Cyn-treated mice displayed a significant reduction in weight loss, colon shortening, and a notably lower DAI score (Figures 1A–D). Furthermore, histological analysis of the colon tissues using H&E staining revealed that the colon tissues of TNBS-induced colitis mice exhibited extensive inflammatory cell infiltration, crypt separation, colon mucosal structural disorganization, hyperplasia, goblet cell loss, and, in severe cases, crypt swelling and ulceration. In contrast, Cyn-treated mice showed reduced inflammatory cell infiltration, significant improvement in crypt separation, mucosal structural integrity, and hyperplasia, along with an increase in goblet cells. There were few crypt abscesses and ulcers, and the inflammation scores were significantly lower than those of the TNBS group (Figures 1E, F). Notably, the levels of inflammatory cytokines (IL-1β, TNF-α, and IL-6) in the colon mucosa of Cyn-treated mice were significantly lower than those in the TNBS group (Figure 1G). Additionally, qRT-PCR analysis demonstrated that Cyn treatment resulted in reduced mRNA expression of TNF-α, IL-6, and IL-1β in the colon mucosa (Figure 1H). These results suggest that Cyn protects mice from TNBS-induced colitis.

### 3.2 Cyn enhances the intestinal barrier’s functionality in mice with TNBS-induced colitis

**FIGURE 2:**
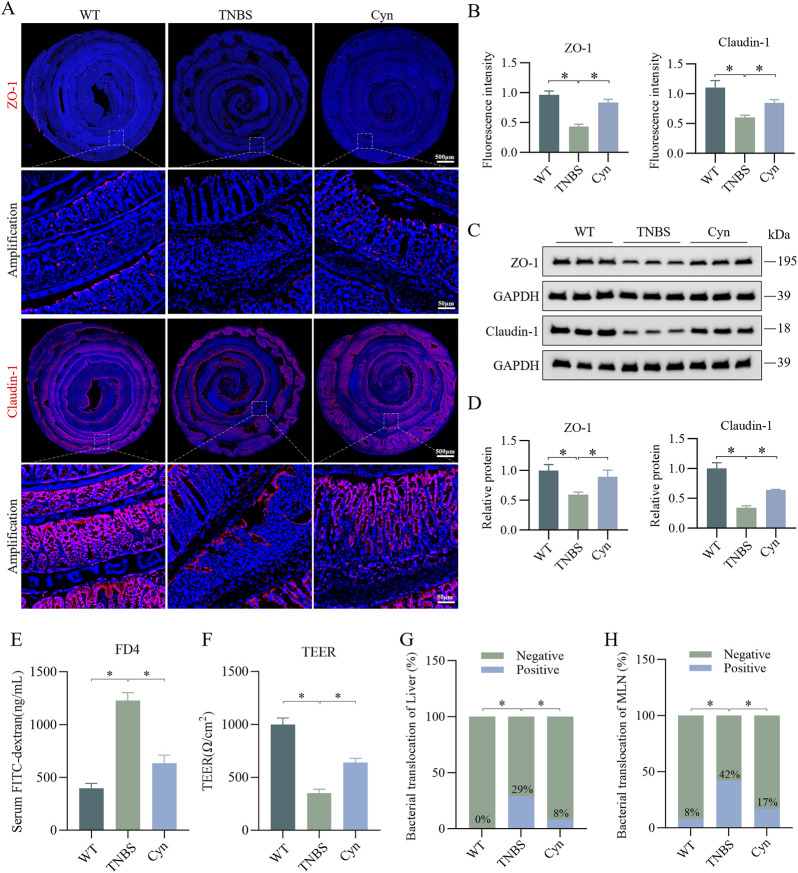
Cyn improves intestinal barrier function in mice with TNBS-induced colitis. **(A)** Immunofluorescence staining of ZO-1 and Claudin-1 in the colon of mice. **(B)** Relative quantification of immunofluorescence staining. **(C)** Western blotting analysis of ZO-1 and Claudin-1 in colon mucosa tissue from mice. **(D)** Relative quantification of protein levels. **(E)** Detection of FITC-dextran (FD4) in mouse blood absorbed into the bloodstream from the intestine. **(F)** Measurement of TEER values in the mouse colon. **(G, H)** Detection of intestinal bacterial translocation in mice. Data are presented as means ± standard deviations, n = 6, *p < 0.05.

The structure and function of the intestinal barrier play a crucial role in the development of CD, with intestinal epithelial tight junction proteins being vital for maintaining barrier integrity (Singh et al., 2019). Immunofluorescence staining showed that Cyn significantly increased the expression of tight junction (TJ) proteins (ZO-1 and Claudin-1) in the colon tissues of TNBS-treated mice (Figures 2A, B). This was further supported by Western blot analysis of the colon mucosa, which showed similar results (Figures 2C, D). Additionally, we assessed the impact of Cyn on intestinal barrier function. In colitis models, the inflammatory response leads to dysfunction of intestinal epithelial cells and the disruption of tight junctions, allowing greater permeability to FITC-dextran. The intestinal permeability data revealed that Cyn treatment notably reduced the absorption of FITC-dextran (FD4) into the bloodstream of TNBS-induced mice (Figure 2E). Furthermore, TEER was measured to evaluate the integrity of the epithelial barrier. TEER values reflect the functional status of tight junctions, with higher values indicating better barrier integrity and lower values signifying increased permeability and impaired function. The TEER values in the Cyn-treated group were significantly higher than those in the TNBS group (Figure 2F). Moreover, increased intestinal permeability typically leads to increased bacterial translocation. Compared to the TNBS group, Cyn treatment substantially reduced bacterial translocation from the colon to organs such as the MLNs and liver (Figures 2G, H). These findings suggest that Cyn protects the intestinal barrier of mice from the disruptions induced by TNBS.

### 3.3 Cyn reduces TNF-α-induced damage to the intestinal epithelial barrier in colonic organoids

**FIGURE 3:**
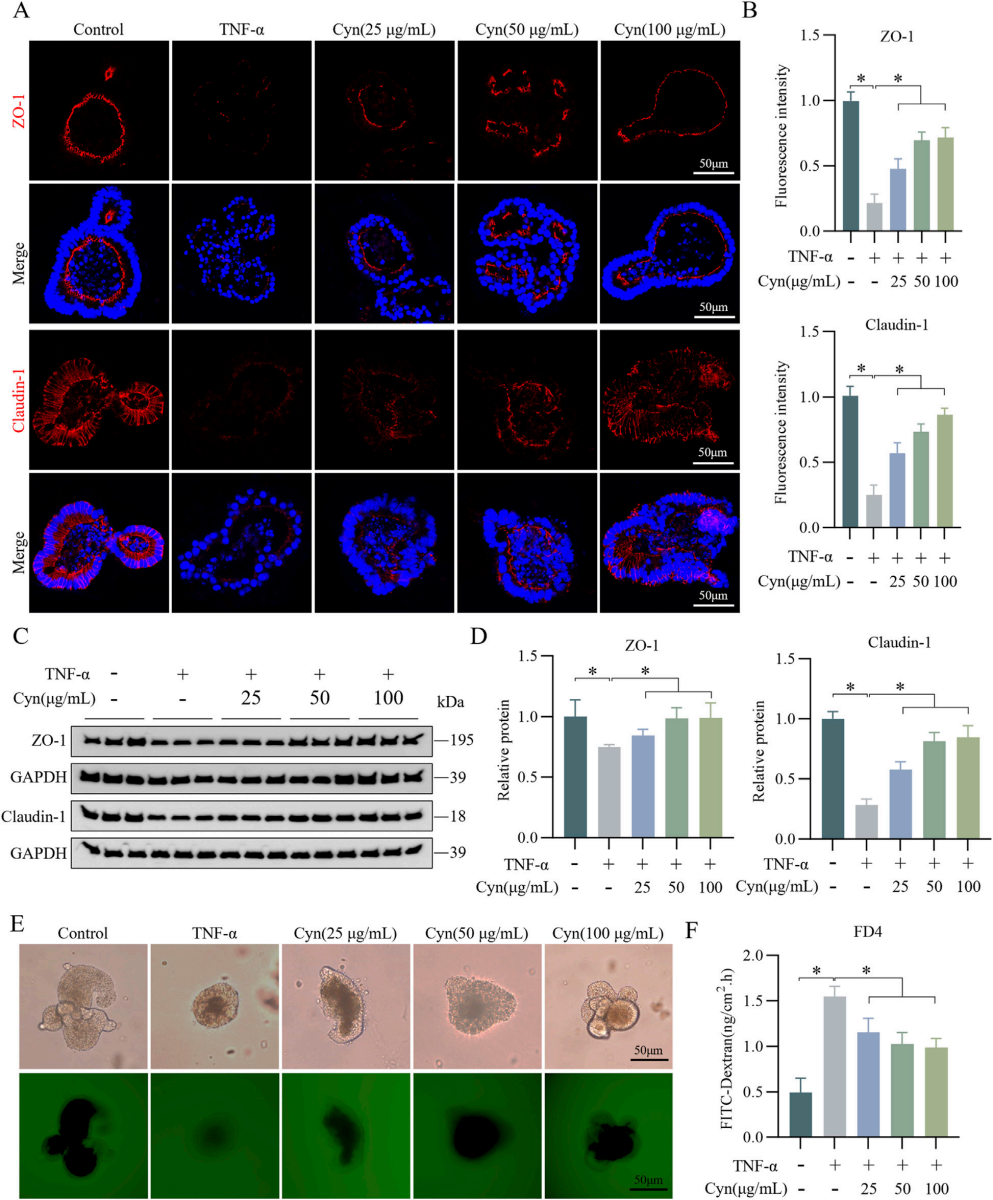
Cyn reduces TNF-α-induced damage to the intestinal epithelial barrier in colonic organoids. **(A)** Immunofluorescence staining of ZO-1 and Claudin-1 in mouse colonic organoids. **(B)** Relative quantification of immunofluorescence staining. **(C)** Protein expression of ZO-1 and Claudin-1 detected by Western blotting in colonic organoids. **(D)** Relative quantification of protein levels. **(E, F)** The permeability of mouse colonic organoids to FITC-dextran (FD4). Data are presented as means ± standard deviations, n = 3, *p < 0.05.

*In vitro*, TNF-α-stimulated mouse colonic organoids were used to create an inflammation model to further investigate whether Cyn protects against intestinal barrier damage. Treatment with various concentrations (0, 12.5, 25, 50, 100, and 200 μg/mL) of Cyn did not significantly affect the activity of the colonic organoids (see Supplementary Figure S2). Cyn treatment effectively prevented the TNF-α-induced reduction in ZO-1 and Claudin-1 expression in the colonic organoids (Figures 3A, B). Western blot analysis of these organoids yielded consistent results (Figures 3C, D). Additionally, permeability assays indicated that Cyn intervention reduced the permeability of colonic organoids to FITC-dextran compared to the TNF-α-treated group (Figures 3E, F). These findings suggest that Cyn protects against TNF-α-induced barrier damage in mouse colonic organoids.

### 3.4 Cyn inhibits IEC apoptosis in TNBS-induced colitis mice

**FIGURE 4:**
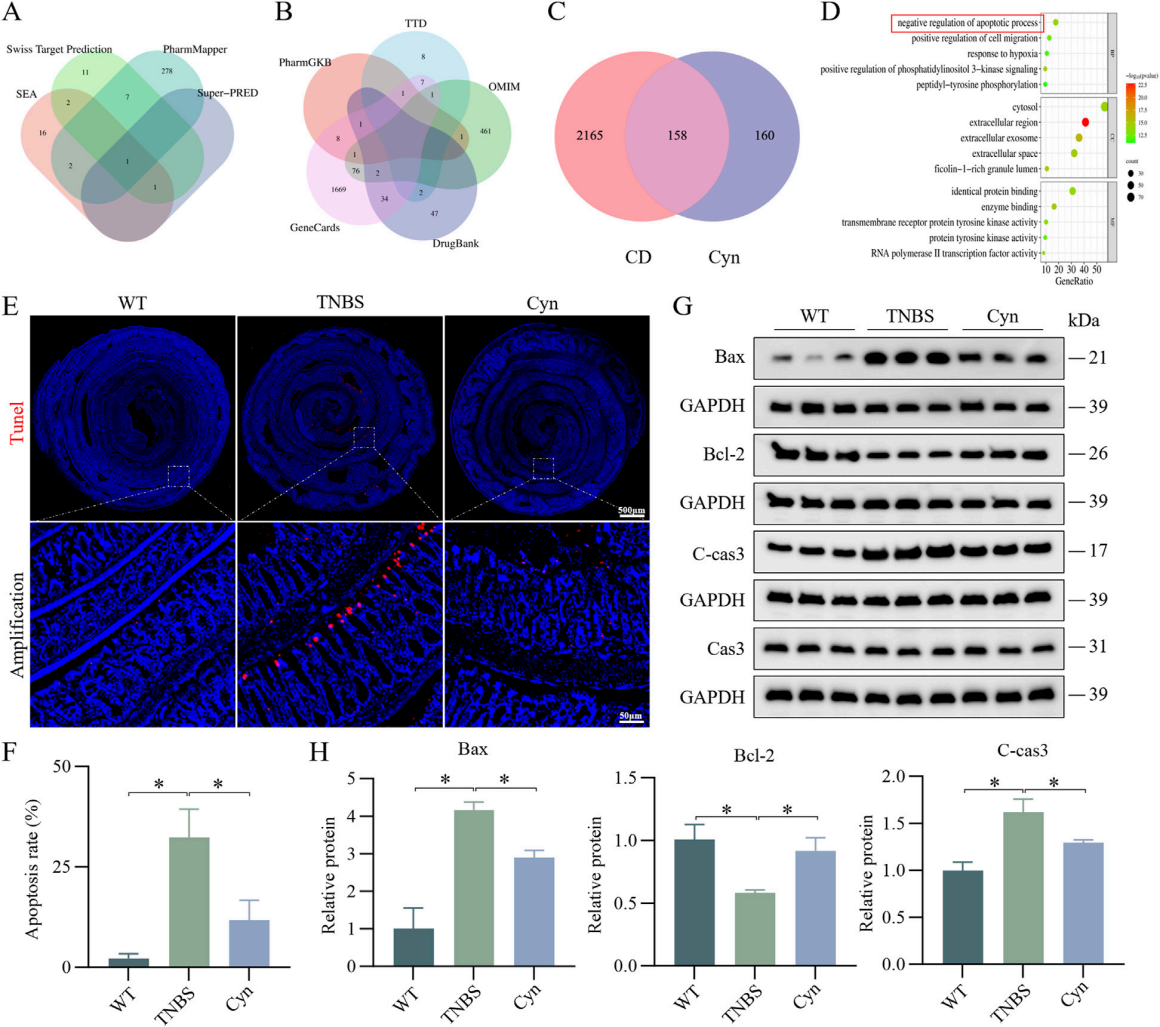
Cyn inhibits IEC apoptosis in TNBS-induced colitis in mice. **(A)** Four databases enriched for target genes related to Cyn. **(B)** Five databases used to enrich target genes relevant to CD. **(C)** Venn diagram showing overlap between Cyn regulatory targets and those involved in CD. **(D)** GO enrichment analysis of cross-talking targets. **(E)** TUNEL staining of mouse colon tissues. **(F)** Positive cell rate determined by TUNEL staining. **(G)** Apoptosis-associated proteins in mouse colon mucosa tissue as determined by Western blotting. **(H)** Relative quantification of protein levels. Data are presented as means ± standard deviations, n = 6, *p < 0.05.

To further explore the biological pathways through which Cyn alleviates colitis and maintains intestinal barrier integrity, a network pharmacology analysis was conducted. A total of 291, 22, 22, and 2 gene targets were identified from the PharmMapper, Swiss Target Prediction, SEA, and Super-PRED databases, respectively. These were converted from protein targets to gene targets via UniProt, resulting in 318 unique Cyn-related genes (Figure 4A). Disease gene targets from GeneCards, PharmGKB, TTD, DrugBank, and OMIM databases included 1800, 12, 19, 86, and 542 genes, respectively, with GeneCards filtered for relevance scores above 6. After eliminating duplicates, 2323 colitis-related target genes were identified (Figure 4B). Of these, 158 common targets were identified as potential Cyn targets for Crohn’s disease treatment (Figure 4C). GO enrichment analysis identified the top five molecular functions (MF), cell components (CC), and biological processes (BP) based on count. These genes were primarily involved in protein hydrolysis and the negative regulation of apoptosis (Figure 4D). To validate whether Cyn affects apoptosis, TUNEL staining showed significantly fewer apoptotic cells in the intestinal epithelium of Cyn-treated mice compared to TNBS-treated mice (Figures 4E, F). Western blot analysis revealed that Cyn treatment upregulated Bcl-2 and downregulated C-cas3 and Bax compared to the TNBS group (Figures 4G, H). These results demonstrate that Cyn prevents colitis and maintains intestinal barrier function by inhibiting epithelial cell apoptosis.

### 3.5 Cyn inhibits TNF-α-induced apoptosis in colonic organoids

**FIGURE 5:**
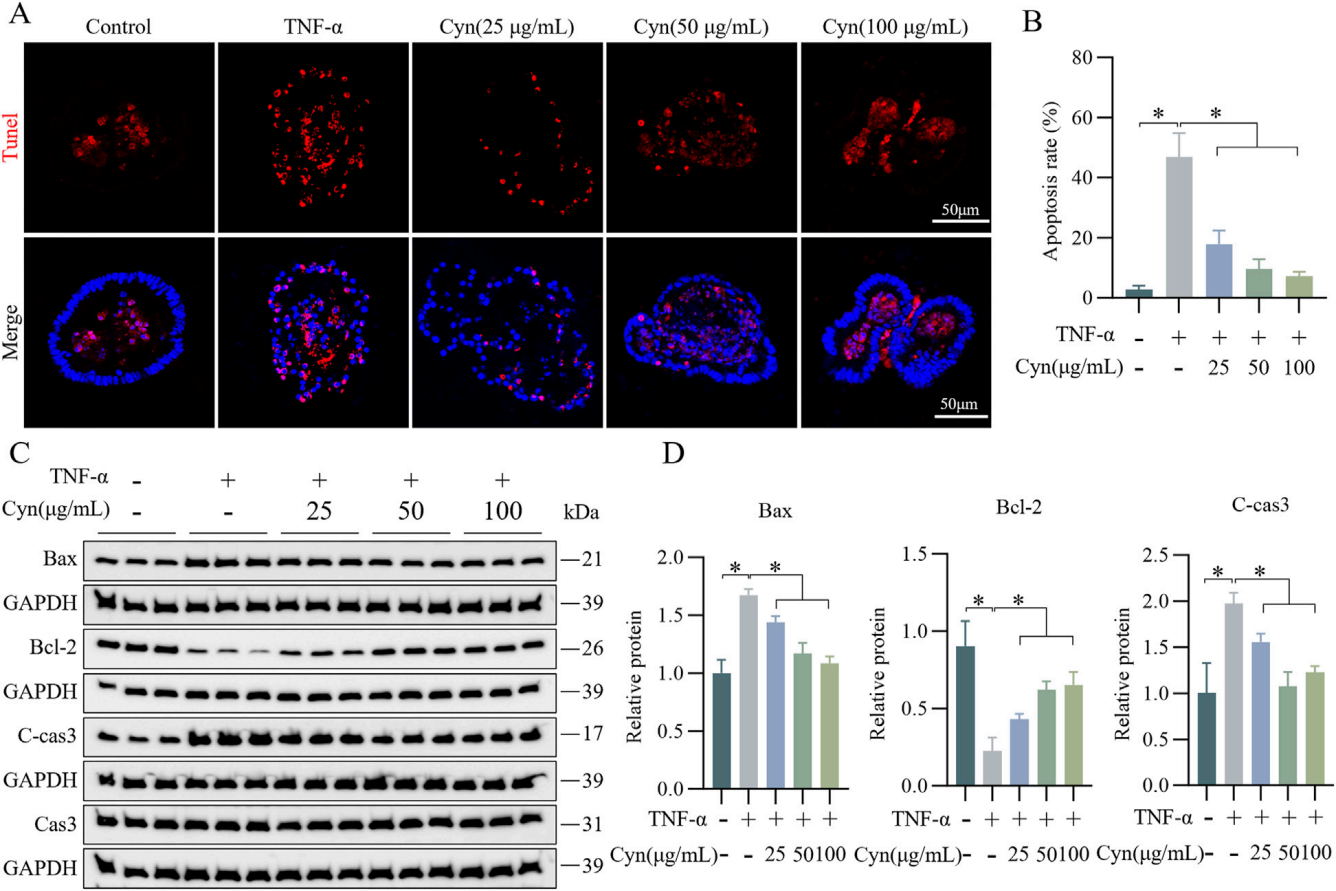
Cyn inhibits TNF-α-induced apoptosis in colonic organoid IECs. **(A)** TUNEL staining of paraffin sections of mouse colonic organoids. **(B)** Statistics for TUNEL-positive cell rate. **(C)** Western blot analysis of apoptosis-related proteins in mouse colonic organoids. **(D)** Relative protein levels. Data are presented as means ± standard deviations, n = 3, *p < 0.05.

Further evidence supporting the idea that Cyn may protect intestinal barrier function by preventing epithelial cell apoptosis was obtained using a mouse colonic organoid model induced by TNF-α. After Cyn treatment, the number of apoptotic epithelial cells in the organoids was much lower than that in the TNF-α-treated group, as observed through TUNEL staining (Figures 5A, B). Additionally, Western blot analysis revealed that, compared to the TNF-α group, Cyn treatment significantly reduced the expression of the apoptosis-related proteins C-cas3 and Bax in colonic organoids, while Bcl-2 expression was significantly increased (Figures 5C, D). Collectively, these findings suggest that Cyn preserves intestinal barrier function by preventing TNF-α-induced apoptosis in colonic organoid epithelial cells.

### 3.6 Cyn’s inhibition of IEC apoptosis is associated with modifications in the PI3K/AKT signaling pathway

**FIGURE 6:**
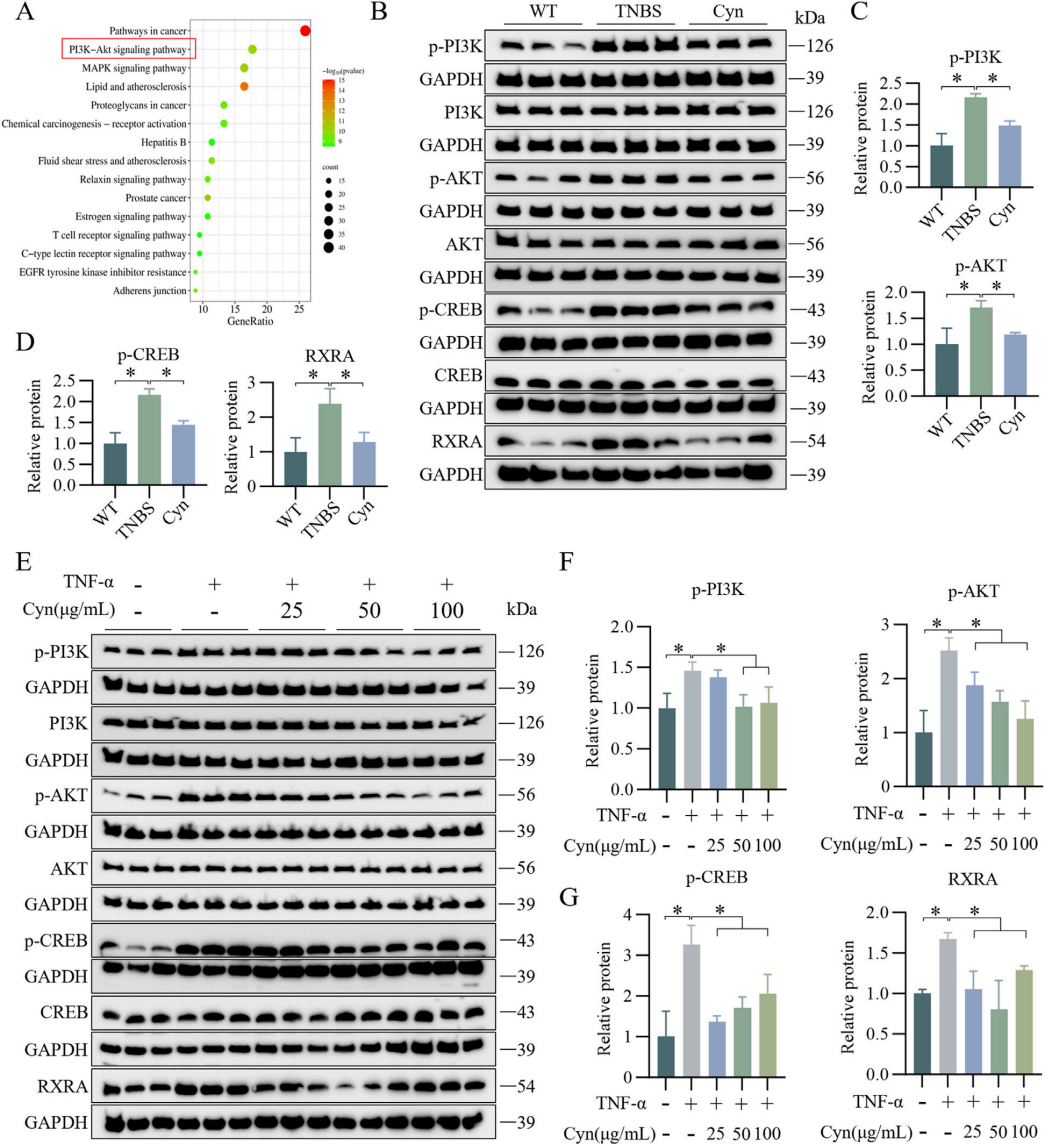
Cyn’s inhibition of IEC apoptosis is linked to changes in the PI3K/AKT signalling cascade. **(A)** KEGG analysis. **(B)** Protein levels (p-PI3K, PI3K, p-AKT, AKT, p-CREB, CREB, and RXRA) in the mucosa tissues of mouse colon examined by Western blotting. **(C, D)** Relative quantification of phosphorylated protein levels. **(E)** Western blot analysis (p-PI3K, PI3K, p-AKT, AKT, p-CREB, CREB, and RXRA) in mouse colonic organoids. **(F, G)** Relative quantification of phosphorylated protein levels. Data are presented as means ± standard deviations, n = 6 *in vivo* or n = 3 *in vitro*, *p < 0.05.

To investigate the mechanisms through which Cyn prevents IEC apoptosis and protects the intestinal barrier, KEGG enrichment analysis was conducted, highlighting gene-related pathways. The top 15 pathways were selected based on gene count. PI3K/Akt signaling was enriched for the largest number of genes (Figure 6A). Moreover, Western blot analysis revealed that Cyn treatment significantly reduced the phosphorylation levels of p-PI3K, p-AKT, p-CREB, and RXRA-key proteins involved in apoptosis within this pathway-in the colon mucosa of TNBS-induced mice (Figures 6B–D). Consistent results were obtained *in vitro* (Figures 6E–G). These findings suggest that Cyn’s protective effect in TNBS-induced colitis is linked to the PI3K/AKT signaling pathway.

### 3.7 Cyn suppresses IEC apoptosis associated with the inhibition of PI3K/AKT signalling in TNBS mice

**FIGURE 7:**
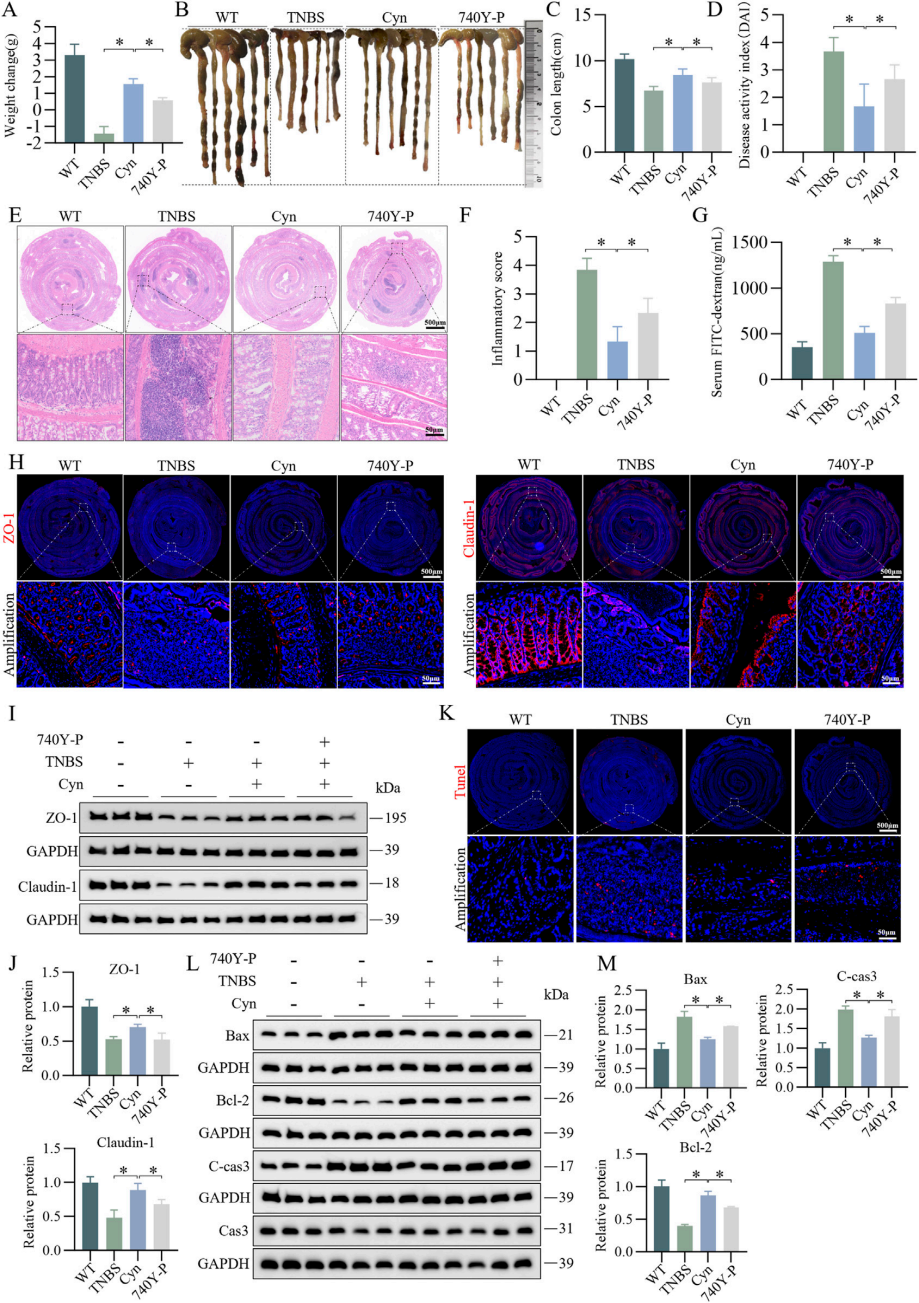
Cyn suppresses IEC apoptosis through inhibition of the PI3K/AKT signalling pathway in TNBS mice. **(A)** Changes in mouse weight. **(B, C)** Appearance of mouse colon and colon length. **(D)** DAI scores. **(E, F)** Colon inflammation scores and H&E staining for each mouse group. **(G)** FITC-dextran (FD4) levels in mouse blood. **(H)** Immunofluorescence analysis of ZO-1 and Claudin-1 in mouse colon tissues. **(I, J)** Western blot analysis of ZO-1 and Claudin-1 in mouse colon mucosa tissues, with relative quantification of protein levels. **(K)** TUNEL staining of colon tissues from mice. **(L, M)** Western blot analysis of apoptosis-related proteins in mouse colon mucosa tissues, with relative quantification of protein levels. Data are presented as means ± standard deviations, n = 6, *p < 0.05.

To determine whether Cyn inhibits IEC apoptosis through a PI3K/AKT-dependent pathway, we administered 740Y-P (10 mg/kg), a cell-permeable PI3K activator that specifically stimulates mitosis (Sun et al., 2020), *in vivo*. The 740Y-P intervention led to significant weight loss (Figure 7A), an increase in colon length (Figures 7B, C), and an increment in DAI scores (Figure 7D) compared to Cyn treatment. Moreover, histological inflammation scores of the colon were significantly higher after 740Y-P intervention than after Cyn treatment, as indicated by H&E staining (Figures 7E, F). In addition, 740Y-P attenuated Cyn’s ability to reduce TNBS-induced intestinal permeability (Figure 7G). Immunofluorescence and Western blot analysis showed that 740Y-P treatment reduced ZO-1 and Claudin-1 expression compared to Cyn therapy (Figures 7H–J). Furthermore, TUNEL staining revealed a substantial increase in IEC apoptosis with 740Y-P treatment compared to Cyn treatment (Figure 7K). Additionally, Western blotting showed that, after 740Y-P intervention, C-cas3 and Bax expressions were upregulated, while Bcl-2 expression was downregulated in the colon mucosa compared to Cyn treatment (Figures 7L, M). In conclusion, Cyn improves intestinal barrier function by partially suppressing intestinal epithelial apoptosis in TNBS-treated mice, at least in part, through inhibition of the PI3K/AKT signaling pathway.

### 3.8 Cyn reduces IEC apoptosis in TNF-α-stimulated colonic organoids associated with the suppression of PI3K/AKT signalling

**FIGURE 8:**
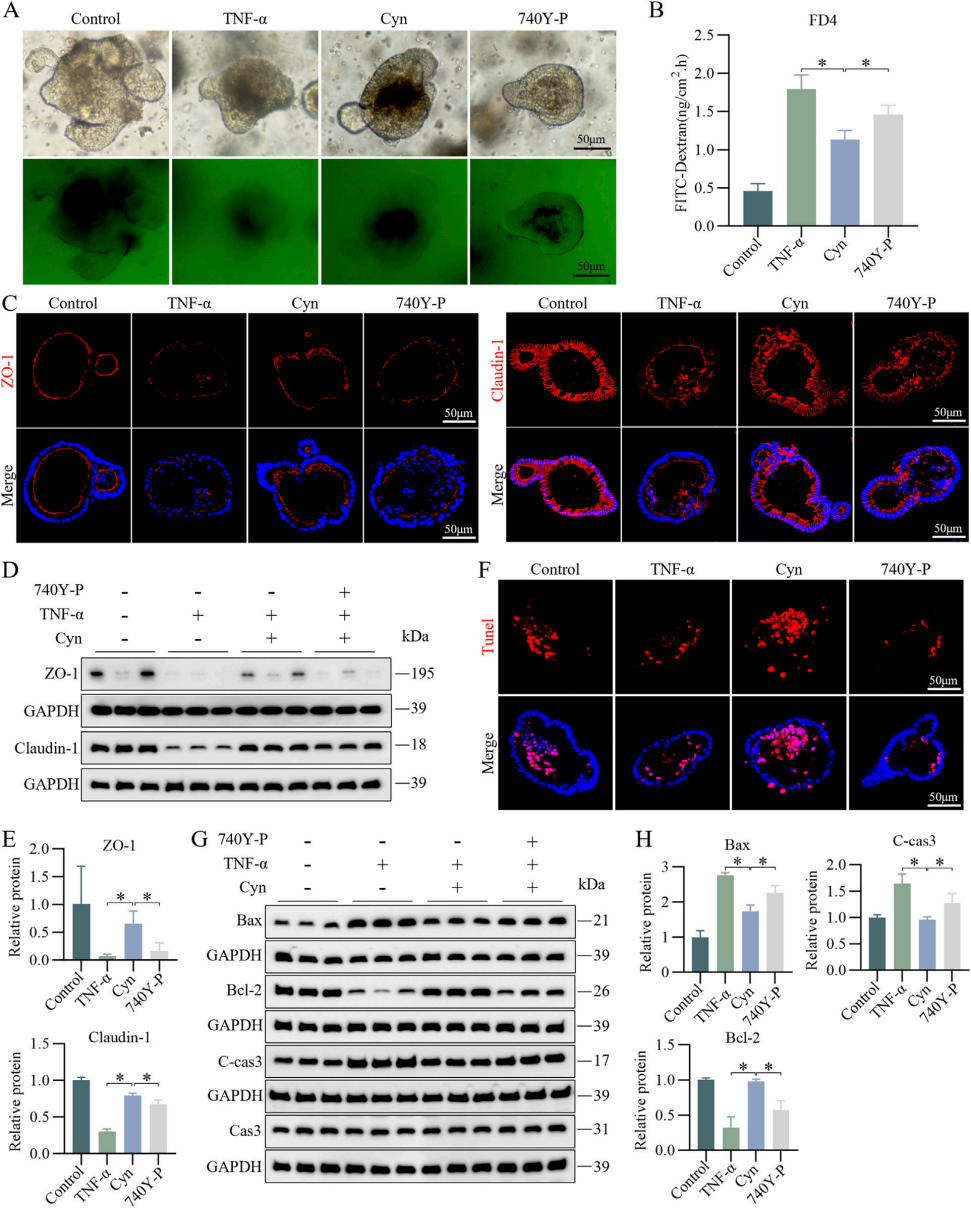
Cyn reduces IEC apoptosis in TNF-α-stimulated colonic organoids through suppression of the PI3K/AKT signalling pathway. **(A, B)** FITC-dextran (FD4) analysis in mouse colonic organoids. **(C)** Immunofluorescence analysis of ZO-1 and Claudin-1 in mouse colonic organoids. **(D, E)** Western blotting analysis of ZO-1 and Claudin-1 levels in mouse colonic organoids and relative quantification of protein levels. **(F)** TUNEL staining of colonic organoids in mice. **(G, H)** Western blotting analysis of apoptosis-related proteins in mouse colonic organoids and relative quantification of protein levels. The data are displayed as means ± standard deviations, n = 3, *p < 0.05.

To further explore whether Cyn reduces apoptosis in IECs through suppression of the PI3K/AKT signaling pathway, we validated this *in vitro* using its activator 740Y-P (20 μM) (Li et al., 2024) in a colonic organoid model. Permeability assays indicated that 740Y-P intervention increased the permeability of colonic organoids to FITC-dextran compared to Cyn treatment (Figures 8A, B). Immunofluorescence results showed that 740Y-P significantly reduced the protein levels of ZO-1 and Claudin-1 in the intestinal epithelium of colonic organoids compared to Cyn treatment (Figure 8C). Western blotting confirmed these findings (Figures 8D, E). Furthermore, TUNEL staining revealed a significant increase in apoptotic IECs in colonic organoids following 740Y-P treatment compared to Cyn treatment (Figure 8F). Additionally, Western blot analysis showed that after 740Y-P intervention, the expression of C-cas3 and Bax was upregulated, while Bcl-2 expression was downregulated, compared to colonic organoids treated with Cyn (Figures 8G, H). Taken together, these results suggest that Cyn inhibits the PI3K/AKT signaling pathway in TNF-induced colonic organoids, thereby partially reducing intestinal epithelial apoptosis.

### 3.9 Cyn exhibits similar inhibition of IEC apoptosis as PI3K/AKT inhibitors in TNBS mice and TNF-induced colonic organoids

**FIGURE 9:**
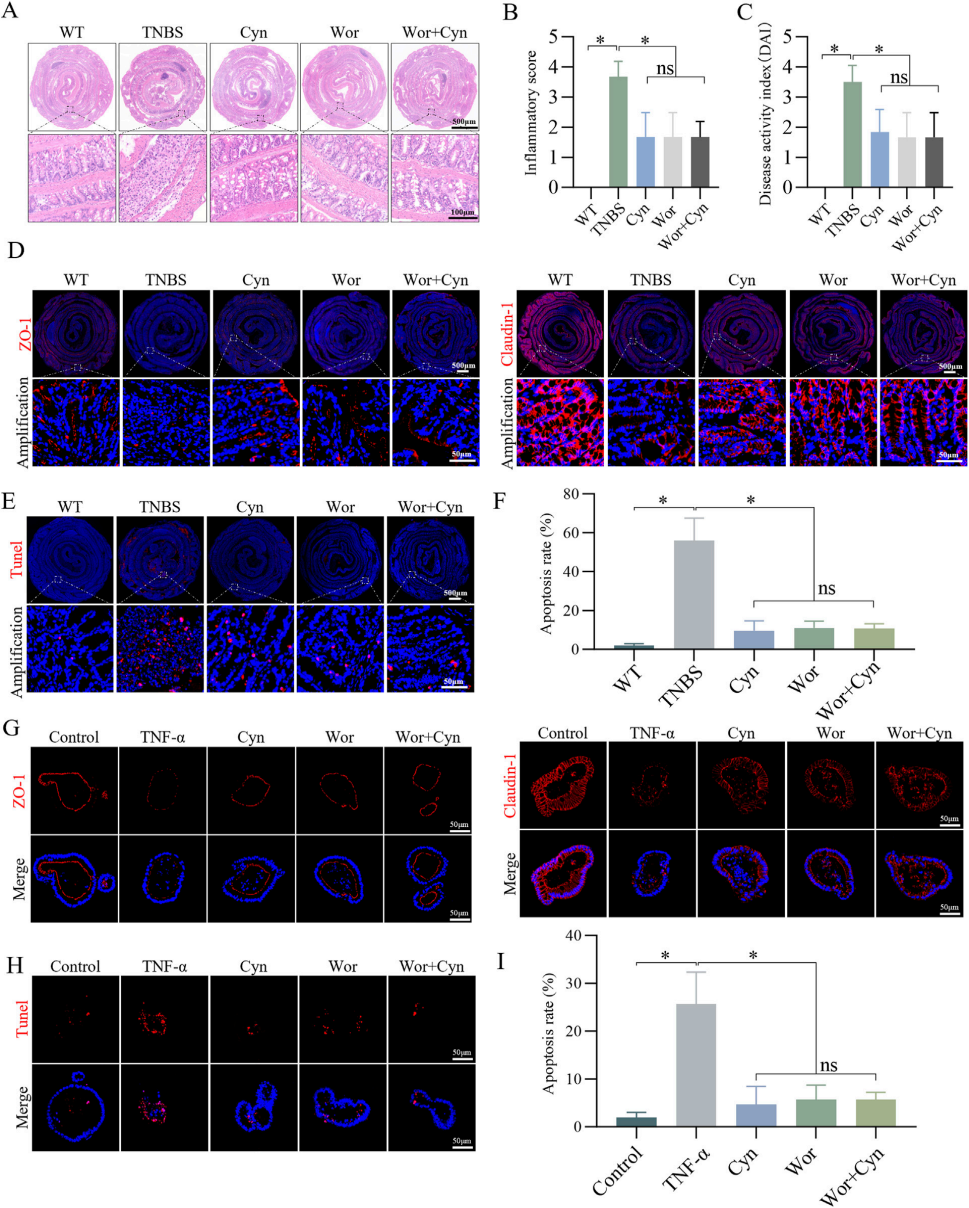
Cyn exhibits a similar inhibition of IEC apoptosis as PI3K/AKT inhibitors in TNBS mice and TNF-induced colonic organoids. **(A, B)** Colon inflammation scores and H&E staining for each mouse group. **(C)** DAI scores. **(D)** Immunofluorescence analysis of ZO-1 and Claudin-1 in mouse colon tissues. **(E, F)** TUNEL staining of colon tissues from mice. **(G)** Immunofluorescence analysis of ZO-1 and Claudin-1 in mouse colonic organoids. **(H, I)** TUNEL staining of colonic organoids in mice. The data are displayed as means ± standard deviations, n = 6 *in vivo* or n = 3 *in vitro*, *p < 0.05, ns: no significance.

To further clarify the role of Cyn in inhibiting PI3K/AKT signaling in IEC apoptosis, barrier damage, and colitis, we used Wortmannin, a potent, selective, and irreversible PI3K inhibitor (Moon et al., 2015). Based on the DAI scores and intestinal tissue inflammation scores derived from H&E staining, Cyn demonstrated anti-colitis effects comparable to those of Wortmannin. Importantly, the combined administration of both drugs did not result in a significant enhancement of their individual therapeutic effects (Figures 9A–C). Additionally, immunofluorescence analysis of ZO-1 and Claudin-1 expression, along with the detection of TUNEL-positive apoptotic cells, revealed that Cyn and Wortmannin similarly reduced apoptosis of intestinal epithelial cells and intestinal barrier damage in in vivo models (Figures 9D–F). Furthermore, Cyn exhibited inhibitory effects on barrier damage (Figure 9G) and IEC apoptosis (Figures 9H, I) comparable to those of PI3K/AKT pathway inhibitors in the TNF-α-induced inflammatory injury model of colonic organoids. These findings collectively confirm that Cyn inhibits IEC apoptosis in a manner similar to PI3K/AKT inhibitors in both TNBS mice and TNF-induced colonic organoids.

## 4 Discussion

Patients with CD often rely on long-term medications, which impose significant social and economic burdens. However, these treatments frequently come with toxic side effects, making them unsustainable for many individuals (Na and Moon, 2019; D'Amico et al., 2022; De Conno et al., 2022; Liu et al., 2023). Consequently, there is an urgent need to identify affordable, safe, and effective therapeutic agents for clinical use. Recently, an increasing number of CD patients have turned to natural remedies derived from plants and herbs (Yang et al., 2021; Yuan et al., 2022). In this study, we investigated the role and mechanism of Cyn, a natural plant extract, in TNBS-induced colitis and intestinal barrier function. Our findings suggest that by inhibiting IEC apoptosis, Cyn reduces colitis and preserves intestinal barrier integrity through the PI3K/AKT signaling pathway.

Using a mouse model of CD, we explored the effects of Cyn in reversing colitis and maintaining intestinal barrier integrity, and the results aligned with our hypotheses. In this study, Cyn alleviated colitis in TNBS model mice through various outcomes, such as increased body weight and colon length, decreased DAI scores, reduced histological inflammation scores, and lower expression of proinflammatory mediators. Impaired intestinal barrier function is a hallmark feature of CD (Noth et al., 2012; Schoultz and Keita, 2019). A growing body of research suggests that both dysfunction and disruption of the intestinal barrier contribute to the development of colitis in CD patients. To investigate this, we used TNBS model mice and TNF-α-induced colonic organoids, where TJ proteins were found to be crucial for maintaining epithelial barrier integrity (Chelakkot et al., 2018). Moreover, recent studies involving the chemotherapeutic drug methotrexate (MTX)-induced intestinal inflammation in rats further confirmed that Cyn alleviates intestinal inflammatory injury (Lang et al., 2024). Our findings revealed that Cyn significantly increased the expression of TJ proteins in both colon tissues and organoids. Intestinal permeability experiments demonstrated that Cyn effectively reduced permeability in both the intestines and organoids. These results suggest that Cyn protects intestinal barrier function by increasing the expression of structural proteins and reducing intestinal permeability. While our study confirms that Cyn helps maintain the integrity of the intestinal barrier and alleviates colitis, the precise mechanism through which it exerts these effects remains to be fully clarified.

The occurrence of apoptosis in the intestinal epithelium is crucial for the continuous renewal of the epithelium and tissue homeostasis. However, an imbalance in apoptosis can lead to increased intestinal permeability and barrier dysfunction (Subramanian et al., 2020). Studies have shown that patients with CD and compromised intestinal barrier function exhibit a higher incidence of IEC apoptosis (Günther et al., 2011; Garcia-Carbonell et al., 2019). Inhibiting IEC apoptosis has been identified as a potential strategy to protect against CD-related intestinal barrier damage. Notably, Cyn has been shown to protect against H_2_O_2_-induced apoptosis in H9c2 cells (Sun et al., 2011), and to reduce apoptosis, mitochondrial dysfunction, and caspase-3 activation in HK-2 cells exposed to cisplatin (Wang et al., 2018). In this study, we also observed that Cyn modulates apoptosis through a network pharmacology approach. Given these findings, we aimed to investigate whether Cyn could protect intestinal barrier function and alleviate colitis by inhibiting IEC apoptosis. First, we confirmed these observations in a mouse model of CD, and the results aligned with our hypothesis. Our study demonstrated that Cyn attenuates IEC apoptosis and reduces intestinal epithelial permeability, while preserving the integrity of the barrier. These results were further supported by experiments using TNF-α-induced mouse colonic organoids, which showed similar outcomes. Collectively, these findings indicate that Cyn can inhibit IEC apoptosis and contribute to the restoration of intestinal barrier function. This suppression of IEC apoptosis prompted us to explore the underlying mechanisms involved.

Network pharmacology is a powerful tool for identifying drugs, their targets, and mechanisms of action. In this study, we utilized network pharmacology to investigate the mechanism of action of Cyn against IEC apoptosis. By analyzing the KEGG pathway enrichment results, we identified the PI3K/AKT pathway as a potential mechanism. It has been reported that the PI3K/AKT signaling pathway plays a key role in regulating IEC apoptosis in CD. For instance, Magnolin blocks the PI3K/AKT pathway, reducing IEC apoptosis and alleviating CD-like colitis (Zhang Z. et al., 2024). Similarly, Ginkgetin suppresses IEC apoptosis through the EGFR/PI3K/AKT pathway, thereby improving experimental colitis (Geng et al., 2024). Based on these findings, we hypothesized that Cyn may enhance intestinal barrier function by inhibiting IEC apoptosis via regulation of the PI3K/AKT signaling pathway. Our data showed that Cyn significantly reduced PI3K and AKT phosphorylation levels, as confirmed by Western blot analysis of colon tissues and organoids from mice. Moreover, in TNBS-induced mice and TNF-α-induced colonic organoids, the inhibitory effect of Cyn on IEC apoptosis and intestinal barrier damage was attenuated by the PI3K/AKT pathway activator 740Y-P. Additionally, inhibition of the PI3K/AKT pathway by Wortmannin resulted in effects on IEC apoptosis and intestinal barrier integrity that were comparable to those observed with Cyn treatment. These findings suggest that Cyn inhibits the PI3K/AKT signaling cascade, reducing IEC apoptosis, protecting the intestinal barrier, and lowering inflammation in TNBS model mice.

In recent years, natural plant extracts have gained significant attention for their use in treating various diseases, largely due to their low toxicity, minimal side effects, resistance to drug tolerance, and stable efficacy. These characteristics make them a promising alternative for addressing the growing need for novel therapeutic agents in clinical practice (Luo et al., 2022; Sun et al., 2022; Wang et al., 2022). Therefore, we sought to identify new alternative treatments that could meet clinical needs. Cyn, a natural plant extract, exhibits a broad range of biological activities (Szekalska et al., 2020; Feng et al., 2021). In addition to its antiapoptotic effects in other disease models (Wang et al., 2018), Cyn has demonstrated significant antiapoptotic effects on IECs in a mouse model of CD, highlighting its potential therapeutic value for treating CD.

Nonetheless, our study has several limitations. First, although we used a TNBS model to simulate CD-like enterocolitis in mice, this model does not fully replicate the clinical symptoms seen in CD patients. Second, while our findings suggest that Cyn alleviates colitis in CD mice by inhibiting IEC apoptosis, other mechanisms may also contribute. Finally, PI3K/Akt is a key signaling pathway known to regulate apoptosis in intestinal epithelial cells, and it is expected to explain the mechanism by which Cyn ameliorates intestinal epithelial apoptosis in colitis mice. However, despite this, we may have overlooked other mechanisms of action of Cyn, such as its role in regulating MAPK signaling.

In conclusion, our research is the first to demonstrate that Cyn, partly through the PI3K/AKT signaling pathway, suppresses IEC apoptosis and alleviates colitis in CD mice. Based on these results, Cyn is expected to target the intestinal barrier and could emerge as a promising alternative therapeutic option for CD in clinical settings. To some extent, this approach also addresses the current challenges in the treatment of CD in clinical practice.

## Data Availability

The original contributions presented in the study are included in the article/Supplementary Material, further inquiries can be directed to the corresponding author.
