# Corrigendum: Cynaroside ameliorates TNBS-induced colitis by inhibiting intestinal epithelial cell apoptosis via the PI3K/AKT signalling pathway

**DOI:** 10.3389/fphar.2025.1591086

**Published:** 2025-03-27

**Authors:** Ju Huang, Jing Li, Zhijun Geng, Lixia Yin, Minzhu Niu, Qingqing Li, Xinyue Liu, Xinke Cheng, Xiaofeng Zhang, Xue Song, Yueyue Wang, Lian Wang, Lugen Zuo, Jianguo Hu

**Affiliations:** ^1^ Department of Clinical Laboratory, First Affiliated Hospital of Bengbu Medical University, Bengbu, Anhui, China; ^2^ Anhui Province Key Laboratory of Basic and Translational Research of Inflammation-Related Diseases, First Affiliated Hospital of Bengbu Medical University, Bengbu, Anhui, China; ^3^ Department of Central Laboratory, First Affiliated Hospital of Bengbu Medical University, Bengbu, Anhui, China; ^4^ Department of Clinical Laboratory, The Third the People’s Hospital of Bengbu, Bengbu, Anhui, China

**Keywords:** Crohn’s disease, cynaroside, IECs apoptosis, colonic organoids, PI3K/AKT

In the published article, there was an error in Figure 7 as published. Two Western blot (WB) strips were misused in Figure 7L (markers: Cas3 and GAPDH). The incorrect strips in Figure 7L were mistakenly used from two strips in **Figure 8G** (markers: Cas3 and GAPDH). The corrected Figure 7 and its caption appear below.

**FIGURE 7:**
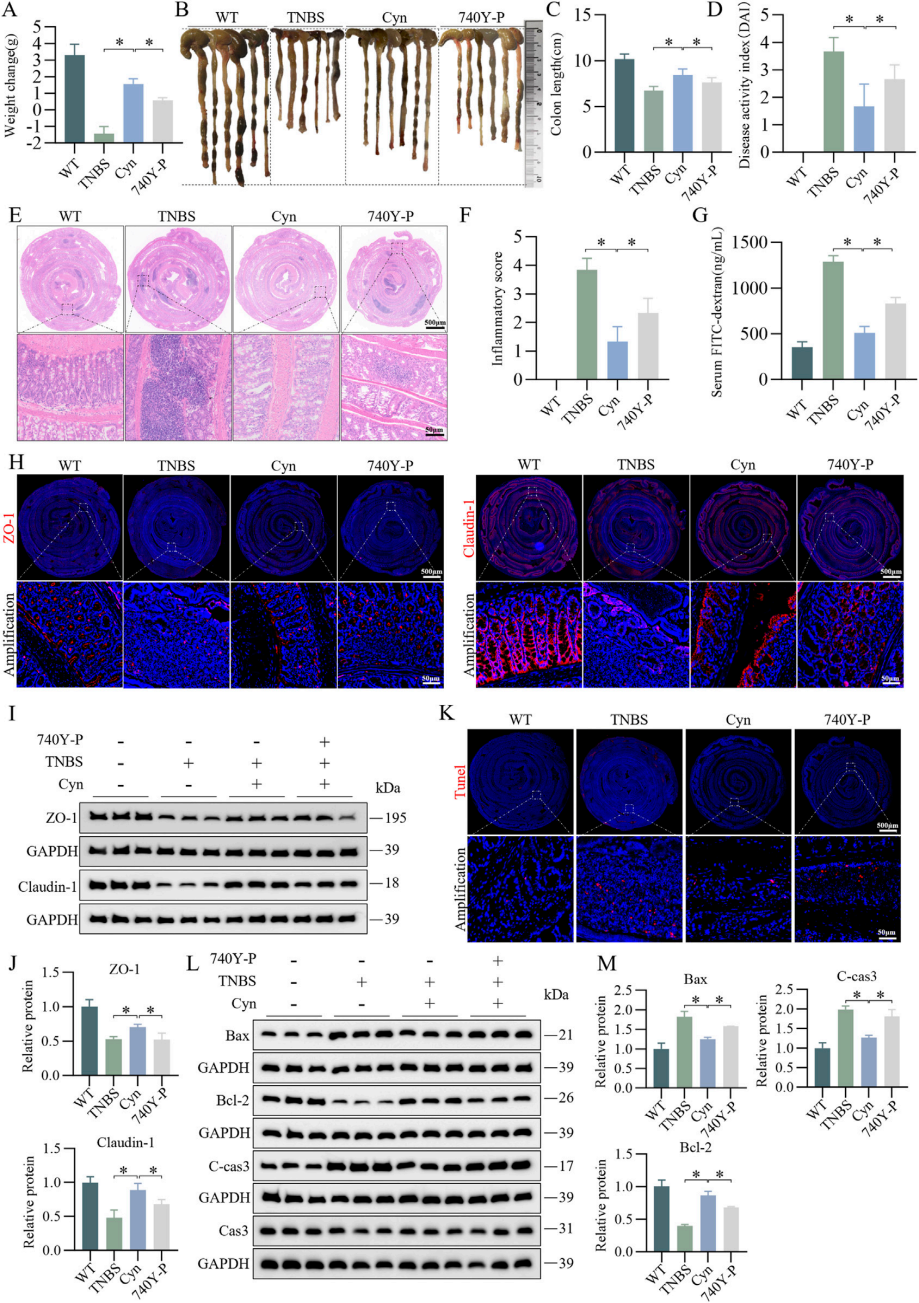
Cyn suppresses IEC apoptosis through inhibition of the PI3K/AKT signalling pathway in TNBS mice. **(A)** Changes in mouse weight. **(B, C)** Appearance of mouse colon and colon length. **(D)** DAI scores. **(E, F)** Colon inflammation scores and H&E staining for each mouse group. **(G)** FITC-dextran (FD4) levels in mouse blood. **(H)** Immunofluorescence analysis of ZO-1 and Claudin-1 in mouse colon tissues. **(I, J)** Western blot analysis of ZO-1 and Claudin-1 in mouse colon mucosa tissues, with relative quantification of protein levels. **(K)** TUNEL staining of colon tissues from mice. **(L, M)** Western blot analysis of apoptosis-related proteins in mouse colon mucosa tissues, with relative quantification of protein levels. Data are presented as means ± standard deviations, n = 6, *p < 0.05.

The authors apologize for this error and state that this does not change the scientific conclusions of the article in any way. The original article has been updated.

