# Supplementary material for: Cynaroside ameliorates TNBS-induced colitis by inhibiting intestinal epithelial cell apoptosis via the PI3K/AKT signalling pathway
Source: Front Pharmacol. 2025 Jan 20;15:1496068. doi: 10.3389/fphar.2024.1496068 (PMC11788346; doi:10.3389/fphar.2024.1496068)
Supplement: Supplementary file 1 [file DataSheet1.pdf]

Supplementary Materials

Supplementary Method 1

Quantitative Assessment of MTT in Vitro

We divided the mouse colon organoids into six groups and administered Cyn at concentrations of 0, 12.5, 25, 50, 100, and 200 μg/mL, Assessment of organoid activity was performed by MTT assay as described previously (Grabinger et al., 2014). It is briefly described as follows: unlike the MTT of conventional cells, after the MTT staining is completed, Matrigel is dissolved in 2% SDS and then tetrazolium salt crystals are dissolved in DMSO, and the absorbance value is measured at 562 nm, and organoid activity is assessed by determining the degree of decrease in MTT.

Supplementary Table 1

Supplementary Table 1 Primer sequences (5′ to 3′)

Gene name	Forward primer	Reverse primer
IL-1β	GAAATGCCACCTTTTGACAGTG	TGGATGCTCTCATCAGGACAG
TNF-α	CAGGCGGTGCCTATGTCTC	CGATCACCCCGAAGTTCAGTAG
IL-6	TCTATACCACTTCACAAGTCGGA	GAATTGCCATTGCACAACTCTTT
GAPDH	TGGCCTTCCGTGTTCCTAC	GAGTTGCTGTTGAAGTCGCA

Supplementary Figures

Supplementary Fig S1

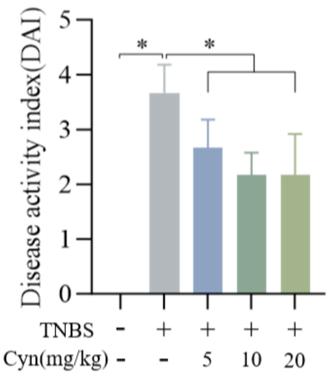

Supplementary Figure 1. DAI scores of a mouse model of TNBS-induced CD-like colitis at different Cyn doses. DAI scores were assigned to each group of mice. The data are presented as the means ± SDs (n=6). *P<0.05.

Supplementary Fig S2

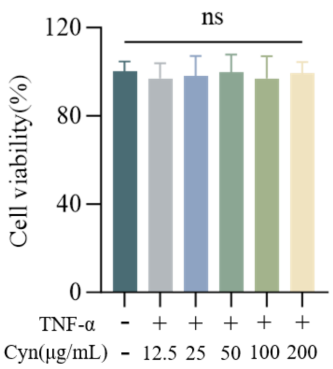

Supplementary Figure 2. Determination of the activity of colon organoids in the presence of different concentrations of Cyn. MTT assay for mouse colon organoid activity. The data are presented as the mean ± SD (n=3), ns: no significance.

Supplementary Figure 3. Uncropped images of blots

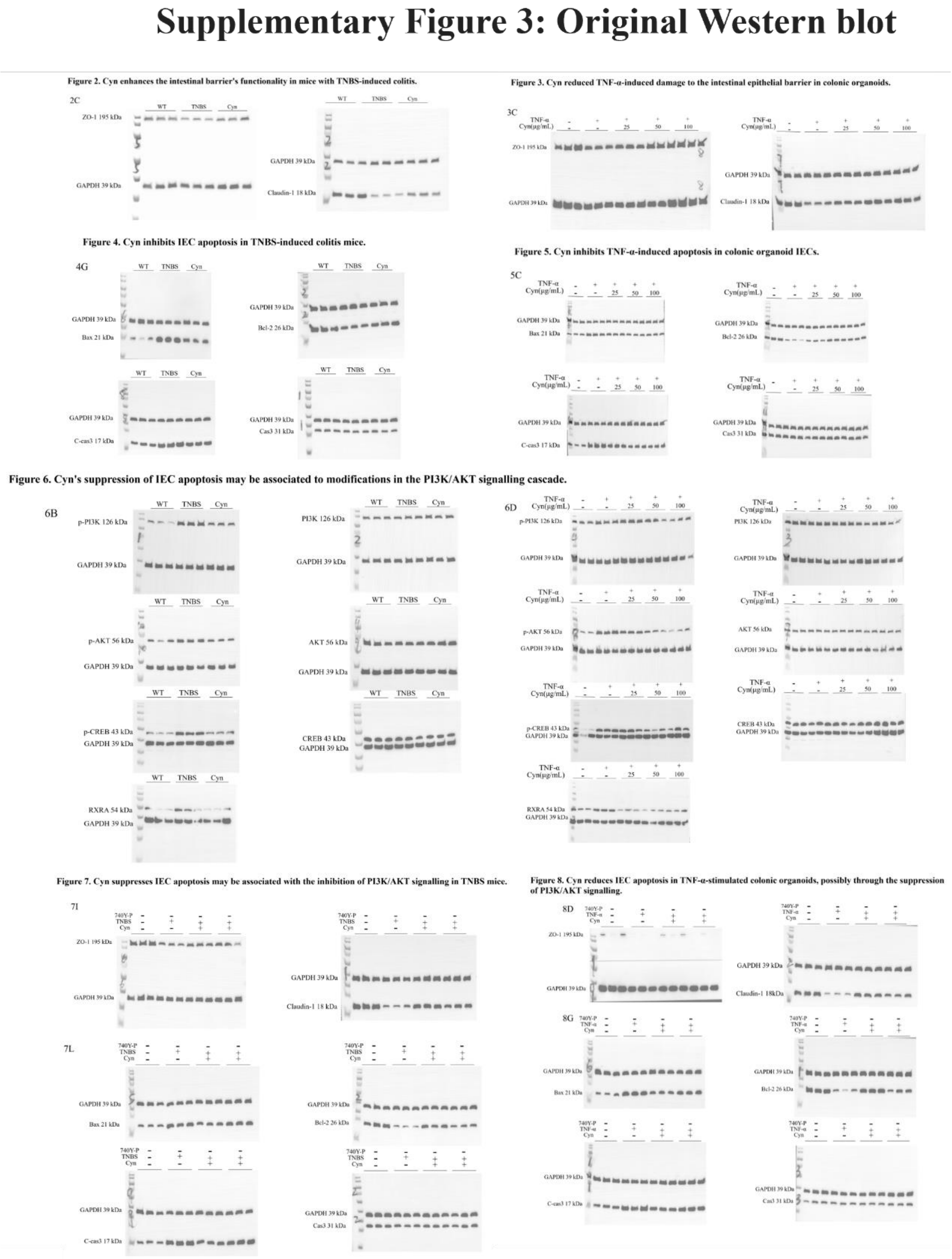

REFERENCES

Grabinger, T., Luks, L., Kostadinova, F., Zimberlin, C., Medema, J. P., Leist, M., et al. (2014). Ex vivo culture of intestinal crypt organoids as a model system for assessing cell death induction in intestinal epithelial cells and enteropathy. *Cell Death Dis* 5, e1228. doi: 10.1038/cddis.2014.183
